# (Ultra-)inflammation or adaptation? Comparison of different ultramarathon distances and their effect on the immune system

**DOI:** 10.3389/fimmu.2026.1799887

**Published:** 2026-04-13

**Authors:** Daniel A Bizjak, Lucas John, Moritz Munk, Roman Bizjak, Jens Witzel, Christoph Siebers, Sebastian V. W. Schulz, Harald Engler, Michael Siebers, Johannes Kirsten, Marijke Grau

**Affiliations:** 1 Department of Internal Medicine, Division of Sports and Rehabilitation Medicine, University Hospital Ulm, Ulm, Germany; 2 Department of Central IT, Division Applications and Databases, University of Zurich, Zurich, Switzerland; 3 Institute for Forensic Psychiatry and Sexual Research, University of Duisburg-Essen, Essen, Germany; 4 Institute of Medical Psychology and Behavioral Immunobiology, Center for Translational Neuro- and Behavioral Sciences, University Hospital Essen, University of Duisburg-Essen, Essen, Germany; 5 Institute of Cardiovascular Research and Sports Medicine, Molecular and Cellular Sports Medicine, German Sport University Cologne, Cologne, Germany

**Keywords:** endurance exercise, exercise stress, immunology, inflammation, ultramarathon

## Abstract

**Background:**

Ultra-endurance sports like running over several hours or days exhibit great physical, psychological and metabolic strain on the respective athlete. Although the impact of ultramarathon running on the inflammatory/immunological system gained interest in the last years, there is no study that examined the effect of different running distances on inflammatory/immune system responses.

**Methods:**

During a non-stop ultramarathon, blood and saliva samples were collected before (Pre) and after the race (Post), and analyzed for changes in blood cell variables (immune cells leukocytes/thrombocytes), cytokine response (pro- and anti-inflammation: IL-6/IL-10/IL-1ra/IL1-beta/TNF-alpha), and stress-related parameters (CRP as acute phase protein; uric acid for oxidative stress; cortisol/kynurenine for general stress and energy metabolism). Biomarkers were supplemented by a stress-related questionnaire and 1) analyzed for the whole group of finishers (N = 43; 16f/27m) and 2) compared between the respective running distances (100/160.9/230 km).

**Results:**

Leukocyte and thrombocyte count increased Post in all runners, with a more pronounced leukocyte response observed in 100 km vs. 160.9 km. IL-6/IL-10/IL-1ra increased Post in all sub-groups, whereas IL1-beta decreased only in the whole group. Stress/immune response showed an increase of salivary cortisol and CRP in all runners. Sub-group analysis revealed highest cortisol and CRP concentrations in 230 km Post race.

**Conclusions:**

Ultramarathons differ in the physiological strain they impose, with running distance being an important factor. Especially 100 km (faster pace, shorter duration) and 230 km (slower pace, longer duration) runners exhibited distinct inflammatory/immunological responses. Thus, broad generalizations regarding the impact of a given ultramarathon on the immune system and potential post-race infection risk are unwarranted, and individualized guidance is currently more effective.

## Highlights

- Ultramarathon running ranges from 50 km to multi-stage races, resulting in acute immunological and inflammatory responses.
- Shorter non-stop competitions (8–16 hours; faster pace, shorter duration) elicit different stress responses compared to longer competitions (20–37 hours; slower pace, longer duration).
- The longer the distance, the more refined and sensible regeneration recommendation strategies for the individual runner should be the focus in the immediate time frame after the race to reduce the risk of infections or immunological damage.
- The long-term immunological consequences of ultramarathon running, depending on race distance, have not yet been examined and require further well-designed studies.

## 1 Background

Ultramarathon includes all distances above the traditional marathon distance of 42.195 km. This broad definition underlines the versatile nature of this running discipline, ranging from single-stage 50 km runs, multi-stage competitions over several days up to nearly non-stop races through countries or even continents (1). The demands exerted on the running athlete include physiological, psychological, metabolic and orthopedic stress, and depends on the individual performance level, experience, stress resilience and competition intensity (1, 2).

Besides the psychological demands of ultramarathon running, which normally requires mental preparation and in-race motivational strategies (3, 4), the especially physiological load contributes to potentially adverse effects. It is well known that long-distance endurance competitions can increase the risk for upper respiratory tract infections (URTI). Possible mechanisms include decreased secretion of salivary antimicrobial proteins, reactivation of latent viruses or a prolonged anti-inflammatory response after a short-term acute pro-inflammatory effect, commonly known as “open window effect” (5–7). During this time after a competition or unaccustomed high training session, athletes are more prone to follow-up infections and diseases. It has been shown that an acute increase in hematological cells following is evident (8), especially in cells related to the immune system response like leukocytes (9) or platelets (10).

While the competition in long-distance running is stressful for the physiological and immunological system, endurance training per se leads to a higher pathogen resilience compared to non-active individuals (11). The continuous bouts of moderate inflammation during training trigger the adaptation of the immune system, with increases in the expression of anti-oxidative enzymes and radial scavenging (11, 12). Hence, the interplay between adequate loading and sufficient regeneration is the crucial factor of a beneficial immune system adaptation in long-distance running.

One key component in the orchestration of the anti- or pro-inflammatory response after exercise are cytokines. Cytokines are signaling molecules that regulate local and systemic immune responses. Maintaining a balance between pro- and anti-inflammatory cytokines preserves tissue health, while disruption may contribute to immunopathology or may improve the risk of development (13). Previous research showed that cytokines can exert versatile functions depending on their origin: for example, endurance training induces an intramuscular increase of interleukin-6 (IL-6), which has pro-inflammatory effects in the circulatory system, but leads to anti-inflammatory downstream processes in the muscle (13, 14). These cytokines like IL-6, IL-8, and IL-15 (15), also known as myokines, can be understood as positive adaptation processes to the endurance training stimulus (15). Here, IL-6 is one of the best examined myokines in the context of endurance exercise and inflammation: As summarized by Waśkiewicz et al, IL-6 increased substantially immediately after ultramarathon races and returned to baseline within 1 to 2 days, while C-reactive protein (CRP) peaked later and remained elevated for 2 to 3 days. Longer distances correlated with higher IL-6 and CRP levels, but experienced runners showed milder responses, partly due to increased anti-inflammatory cytokines like IL-10, whereas findings on changes in Tumour-Necrosis-Factor-alpha (TNF-α) were inconsistent (16).

With increasing training volume like in ultramarathon running, the possibility of an impaired immune system may concomitantly increase due to shorter regeneration times and more stressful training strain, but evidence in the field of prolonged endurance exercise like ultramarathon running is scarce (17). Further markers like damage associated uric acid or the main downstream metabolite of the tryptophan metabolism kynurenine, which is upregulated, e.g. in mental disorders, immunological stress responses and overtraining syndrome (18, 19), are proposed as potential analytical biomarker to monitor immunological load. As this monitoring remains difficult without a continuous biomarker assessment, it is still under debate if there is a further turning point with increased training and lower infection rate – especially in highly-trained elite-athletes successful for many years (20).

Thus, as there is only limited evidence in the examination of the relationship between ultra-endurance competitions in well-trained athletes and subsequent effect on the athletes’ immune system, we aimed to examine the relationship between ultramarathon running and the immune system response before and after an ultramarathon competition, and to analyze possible different effects depending on competition distance. Therefore, we used a combination of blood (reflecting the circulatory system) and saliva (reflecting oral respiratory mucosal immunity) sampling as well as a questionnaire with sub-categories to immune-related side effects.

## 2 Materials and methods

Data was collected during the ultramarathon TorTour de Ruhr^®^, a non-stop ultramarathon held every two years in Germany which covers the distances 100 km, 160.9 km and 230 km. All participants run on the same course (Ruhr Valley Bike Trail) that follows the river to the Rhine, though they began from different starting points. Before (Pre) and after the race (Post), blood as well as saliva samples were collected for molecular analysis, while validated questionnaires were used to assess training and competition status as well as subjective psychological and physical symptoms.

### 2.1 Entry eligibility

A general prerequisite for the TorTour de Ruhr^®^ participation was a medical sports examination that has been conducted less than 6 months before the race and that confirms the physical capacity of the athlete for a healthy arrival, and a personal invitation of the running competition head to ensure sufficient experience. Depending on the self-selected running distance during registration, all runners had to show proof of ultramarathon experience. This included a successfully finished ultramarathon run for the 100 km (at least 50 km), a 6-hours run or longer for the 160.9 km, and 24-hours running competition for the longest distance of 230 km entry eligibility. General performance characteristics (training load, completed marathon and ultramarathon races) were determined by questionnaires.

Inclusion criteria for the study participation were as follows: male/female; endurance athlete and participant of the TorTour de Ruhr^®^ 2024, covering the distances of either 100 km, 160.9 km or 230 km; no immediately preceding injuries and the ability to understand the study procedure and to give informed consent.

Exclusion criteria included: nicotine consumption; chronic gastrointestinal disorders; blood clotting disorders or acute use of anticoagulants; acute or chronic vascular (blood flow) disorders; cardiovascular, metabolic or autoimmune diseases or non-consenting subjects. No other exclusion criteria were applied besides a missing medical sports examination.

All subjects were provided with information about the content of the study, the use of the data and gave written consent. The study was conducted in compliance with the Declaration of Helsinki. The study was approved by the ethics committee of German Sports University Cologne (12/2024).

### 2.2 Anthropometry, body composition and biosampling

Forty-three (16 female, 27 male) ultramarathon runners were included in the study. Three participants did not reach the required finish cut-off time, but sampling was partly possible and used for analysis if appropriate (anthropometry, questionnaires). Thus, in total, data sets of N = 19 (100 km), N = 8 (160.9 km) and N = 16 (230 km) runners were used for final analysis.

Anthropometric measurements included height, body mass and body composition. Height was measured without shoes, in light clothing with a standardized scale. For measuring body mass and body composition, a bio-impedance scale (InBody 770, InBody Europe B.V., Eschborn, Germany) was used.

The study involved both pre- and post-race assessments. Pre-race measurements were conducted either the evening before the 230 km race (from 5 to 8 pm) or beginning two and a half hours before the start of the 160.9 km race (from 3:30 to 5:30 pm) and the 100 km race (from 1:30 to 3:30 am).

Post-race assessments took place immediately at the finish line upon participants’ arrival, beginning with blood collection (with a delay of approximately 5 minutes). Pre as well as Post race, a total of 21 ml of blood was drawn from the participants’ *vena mediana cubiti* into EDTA-treated blood collection tubes (S-MONOVETTE^®^, Sarstedt, Nümbrecht, Germany). 2.7 ml of drawn blood was used for complete blood count analysis, while the remaining collection tubes were immediately centrifuged at 2000 g for 10 minutes at 4 °C. Plasma was aliquoted into 0.5 ml tubes and initially stored at –20 °C and, within a maximum of six hours, transferred to –80 °C until final analysis. All Post samples were taken in a time frame of 10 minutes after crossing the finish line.

For saliva sampling, all participants had to drool 5 ml of saliva into a 50 ml Falcon tube (Fisher Scientific, Schwerte, Germany) Pre and Post race as fast as possible. Flow rate and sample quality could not be standardized, but drooling had to be finished in the time frame of five minutes. Drinking water ad libitum was allowed. Samples were immediately stored at appropriate temperatures. After thawing, samples were centrifuged at 2370 g for 10 minutes at room temperature and the clear supernatant was aliquoted into 2 ml tubes and stored at -80 °C until analysis.

### 2.3 Blood values

A complete blood count (CBC) was performed by a medical laboratory (Dr. Wisplinghoff, Cologne, Germany). Since only leukocyte and platelet values are relevant for the present research question, only these are reported here. Parameters related to red blood cells (RBCs) will be used for a different research question and are thus excluded from the present study.

### 2.4 Inflammatory markers

Plasma concentrations of TNF-α, IL-6, IL-1ra and IL-10 were measured by multiplex electrochemilumenscence (ECL) assay (U-plex, Meso Scale Discovery, Rockwell, MD, USA) on a MESO QuickPlex SQ 120MM instrument (Meso Scale Discovery) according to the manufacturers’ protocol. Sensitivity of the assay was 0.51 pg/ml for TNF-α (#K251UCK intra-assay CV<3.5%, inter-assay CV<9%), 0.33 pg/ml for IL-6 (#K151TXK intra-assay CV<4%, inter-assay CV<10%), 1.7 pg/ml for IL-1ra (#K151XPK intra-assay CV<4%, inter-assay CV<10%), and 0.14 pg/ml for IL-10 (#K151TZK; intra-assay CV<3.5%, inter-assay CV<10%). Salivary concentrations of CRP (Salimetrics, Carlsbad, CA, USA; #1-2102; intra-assay CV<2%, inter-assay CV<5%), IL1-beta (Salimetrics; #1-3902; intra-assay CV<3%, inter-assay CV<5%) and IL-17A (MyBiosource; #MBS9425042 intra-assay CV<10%, inter-assay CV<12%) were determined by ELISA according to the manufacturers’ instructions. Sensitivity of the assays were 1.79 pg/ml for CRP, 0.37 pg/ml for IL1- beta and 0.74 pg/ml for IL-17A.

### 2.5 Cellular stress response

Kynurenine concentrations were measured spectrometrically in plasma samples based on an established protocol by Bizjak et al. (18). Salivary uric acid (#5421; sensitivity 0.07 mg/dl; intra-assay CV<2%, inter-assay CV<5%) were analysed in saliva samples in accordance with the manufacturers’ instructions via ELISA-technology (Salimetrics, Carlsbad, CA, USA);. Cortisol was determined both in plasma (Thermo Fisher Scientific, Waltham, MA, USA; #EIAHCOR; sensitivity 17.3 pg/ml; intra-assay CV<9%, inter-assay CV<9%) and saliva (Salimetrics, Carlsbad, CA, USA; #1-3002; sensitivity <0.007 µg/dl; intra-assay CV<5%, inter-assay CV<6%) to examine possible differences between these two matrices. All measurements were performed as described in the manufacturers’ manuals.

### 2.6 Questionnaire: general assessment of side effects

The validated General Assessment of Side Effects (GASE, German version) questionnaire was used to assess sides effects occurred during the race. In brief, GASE consists of 25 questions on a 4-point Likert scale, ranging from symptoms regarding nutrition-/pain-/immune-/psychological-related issues, and with the grading from None, Low, Moderate, and High. A total score can be calculated from all answers, with higher scores indicating higher side effects. Data presented in this manuscript contain the analysis of the total score as well as a sub-analysis with regard immune system related side effects (21).

### 2.7 Use of artificial Intelligence in manuscript preparation

The free online version of ChatGPT was solely used as a tool for relevant literature search.

### 2.8 Statistics

Data analysis was performed using GraphPad Prism (GraphPad Prism 10.5; San Diego, CA, USA). Normal distribution of the variables was examined using the Kolmogorov-Smirnov normality test. Paired t-tests were used for comparisons between Pre and Post samples for all normally distributed data. Otherwise, a Wilcoxon matched-pairs signed rank test was used to determine statistical significance of differences between Pre and Post. Differences between the 100 vs 160.9 vs 230 km participants were determined using One-way ANOVA followed by Holm-Šídák’s multiple comparisons test (normally distributed data) or with Kruskal-Wallis test followed by Dunn’s multiple comparisons test (not normally distributed data). As there is a known sex-related differences in immune and inflammatory responses (22), an exploratory analysis was performed between female (N = 16) and male (N = 27) participants using One-way ANOVA followed by Holm-Šídák’s multiple comparisons test (normally distributed data) or with Kruskal-Wallis test followed by Dunn’s multiple comparisons test (not normally distributed data). Effect sizes are reported as partial eta squared ( $\eta_{p}^{2}$), where $\eta_{p}^{2}$=0.01 signifies small, 0.06 $\eta_{p}^{2}$ medium, and 0.14 $\eta_{p}^{2}$ large effects for parametric tests only. For non-parametric Wilcoxon matched-pairs signed rank tests, GraphPad Prism does not compute any effect sizes. If not otherwise stated all data are presented as mean ± standard deviation. Statistical significance was established at p ≤ 0.05.

## 3 Results

### 3.1 Study group characteristics

Detailed anthropometric characteristics of all runners are shown in **Table 1**. Regarding the sub-groups, there was no significant difference either in age, body mass, body height, body fat or skeletal muscle mass between running distances. All participants had a mean ultramarathon experience of 38 successfully finished ultramarathon competitions. Details on subgroup analysis can also be found in the study by John et al. (23) and Grau et al. (24).

**Table 1:** Descriptive statistics of the 100/160.9/230 km participants. Included are information on anthropometry, training/competition experience and finish time.

Participants: n=43 [16f/27m]	Mean ± SD		Range
Age [years]	49.3 ± 8.7		39
Body mass [kg]	71.43 ± 10.58		48.30
Body weight [m]	174.3 ± 6.4		35.4
Body Mass Index [kg/m²]	23.45 ± 2.39		9.7
Skeletal muscle mass [kg]	32.79 ± 5.54		24.50
Body fat mass [kg]	13.08 ± 4.93		19.20
Body fat [%]	18.17 ± 6.09		22
Weekly running distance [km]	71.4		128
Completed marathons	51		178
Completed ultramarathons	38		188
	100	160.9	230
Finish time [hh:mm:ss]	14:47:42	23:21:51	32:49:23

### 3.2 Blood values

**Figure 1:**
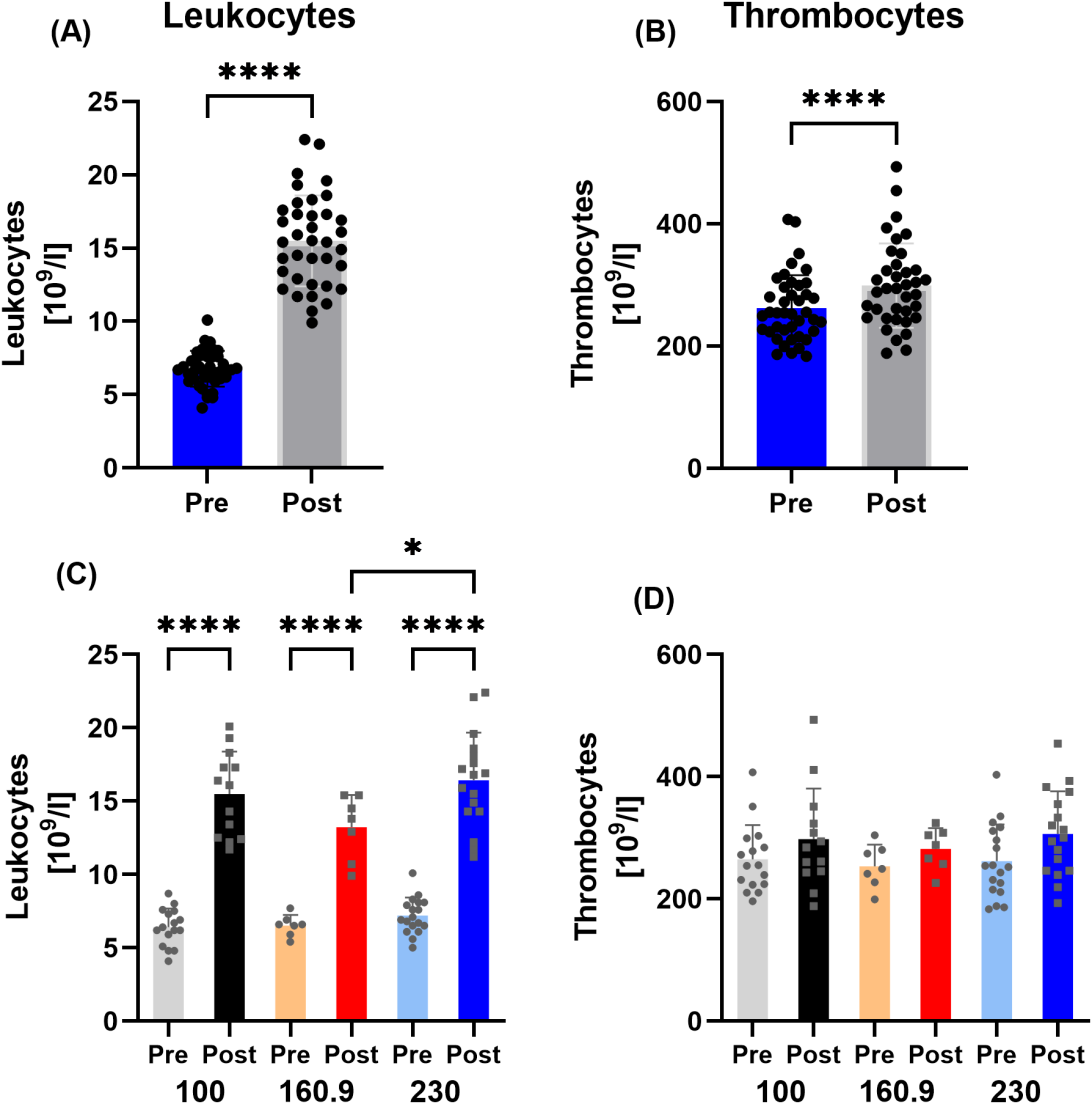
Data on **(A)** leukocyte concentration and **(B)** thrombocyte concentration for all participants, as well as subdivided into the respective running distance of 100/160.9/230 km for **(C)** leukocytes and **(D)** thrombocytes. While there was an increase in leucocytes Post race in all participants and subgroups as well as between 160.9 km and 230 km, thrombocyte cells did only differ between Pre and Post. *p < 0.05; ****p < 0.0001.

The number of leukocytes increased after the race. These findings applied to both the overall analysis and the Post values for each run (p<0.0001, $\eta_{p}^{2}\;$0.9277). Comparison of the Post values showed significantly higher values Post 230 km compared to Post 160.9 km (p=0.0111) (**Figures 1A, C**).

Thrombocyte counts increased in the aggregated Post analysis across all runs (p<0.0001, $\eta_{p}^{2}$0.3735). However, when stratified by individual running distances, no Post increase was observed (**Figures 1B, D**).

### 3.3 Inflammatory markers

**Figure 2:**
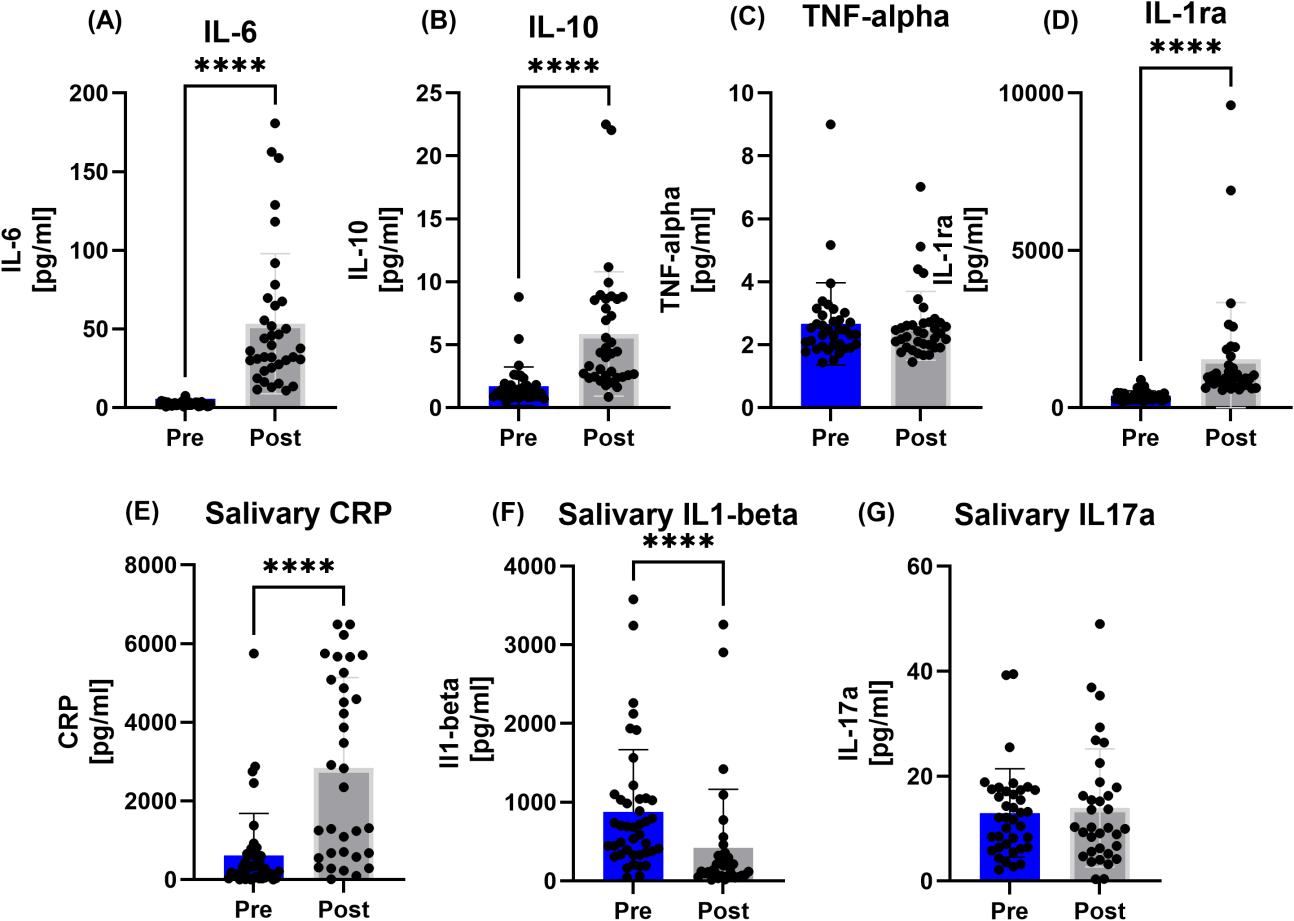
Data on **(A)** interleukin 6 (IL-6), **(B)** interleukin 10 (IL-10), **(C)** tumor-necrose-factor-alpha (TNF-alpha), **(D)** interleukin 1ra (IL-1ra), **(E)** salivary C-reactive protein (CRP), **(F)** salivary interleukin 1-beta (IL1-beta), and **(G)** salivary interleukin 17a (IL17a) concentrations for all participants. While IL1-beta decreased Post race in all participants, the interleukins IL-6, IL-10 and IL-1ra as well as salivary CRP increased after the race. ****p < 0.0001.

Regarding all participants, salivary IL1-beta decreased Post, whereas plasma levels of Il-6, IL-10 as well as IL-1ra showed increased values (p<0.0001). Saliva IL17a and TNF alpha remained unaffected Post, while salivary CRP increased (p<0.0001) (**Figure 2**).

**Figure 3:**
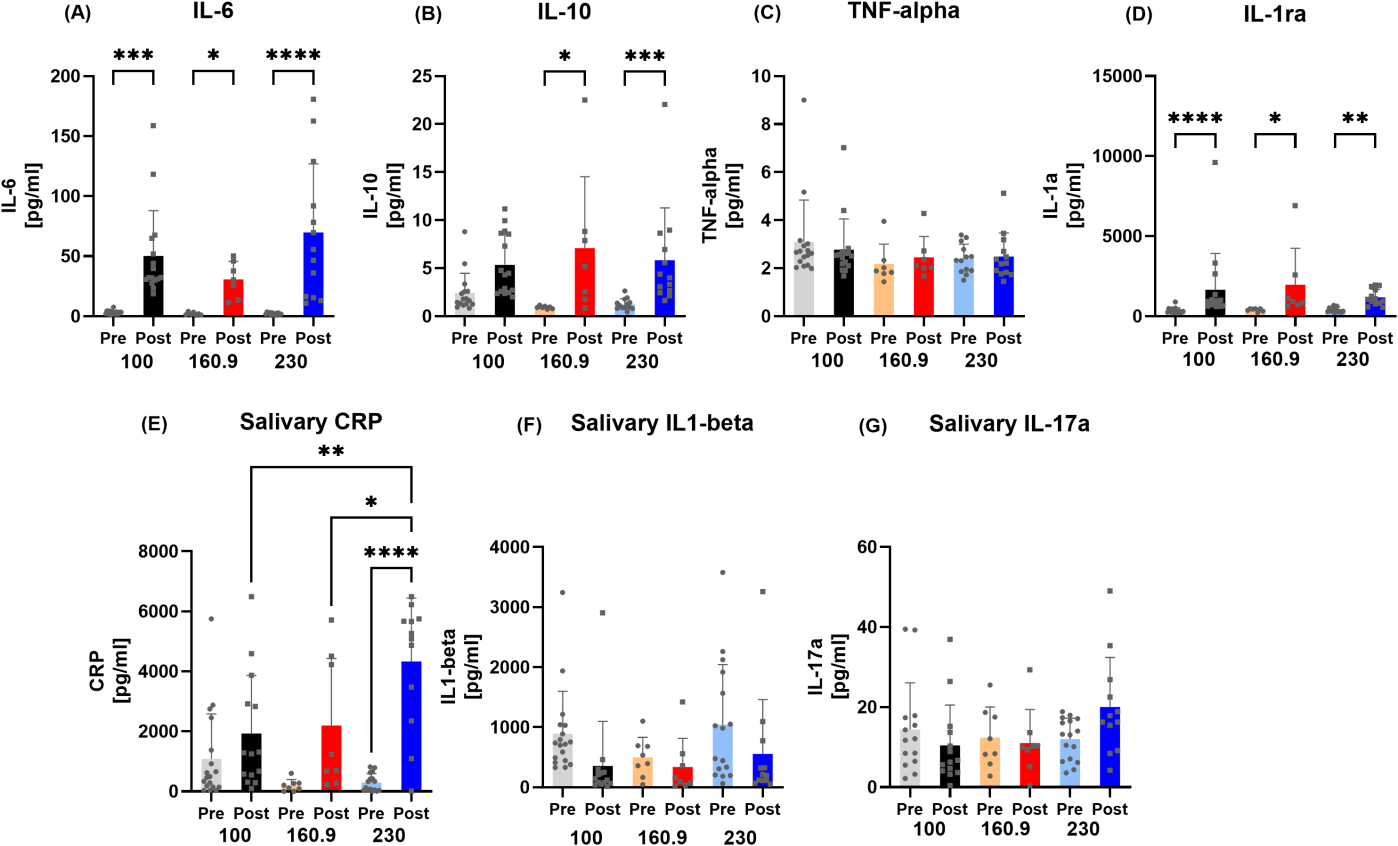
Data on **(A)** interleukin 6 (IL-6), **(B)** interleukin 10 (IL-10), **(C)** tumor-necrose-factor-alpha (TNF-alpha), **(D)** interleukin 1ra (IL-1ra), **(E)** salivary C-reactive protein (CRP), **(F)** salivary interleukin 1-beta (IL1-beta), and **(G)** salivary interleukin 17a (IL17a) concentrations subdivided into 100/160.9/230 km running distance. Except for the 100 km runners regarding IL-10, IL-6, IL-10 and IL-1ra increased in all sub-groups Post race. Salivary CRP concentrations were higher in 230 km compared to the other groups Post race. *p < 0.05; **p < 0.01; ***p < 0.001; ****p < 0.0001.

With respect to the individual distances, plasma interleukins IL-6 (100 km p<0.0003; 160.9 km p=0.0231; 230 km p<0.0001) and IL-1ra (100 km p<0.0001; 160.9 km p=0.0404; 230 km p=0.0011) were elevated in all groups, whereas IL-10 levels increased in the 160.9 km (p=0.0151) and 230 km (p=0.0006) groups, and saliva CRP in the 230 km group (p<0.0001) (**Figure 3**). Moreover, 230 km-CRP concentrations were significantly higher compared to the other distances (100 km p<0.0031; 160.9 km p=0.0445).

### 3.4 Stress response

**Figure 4:**
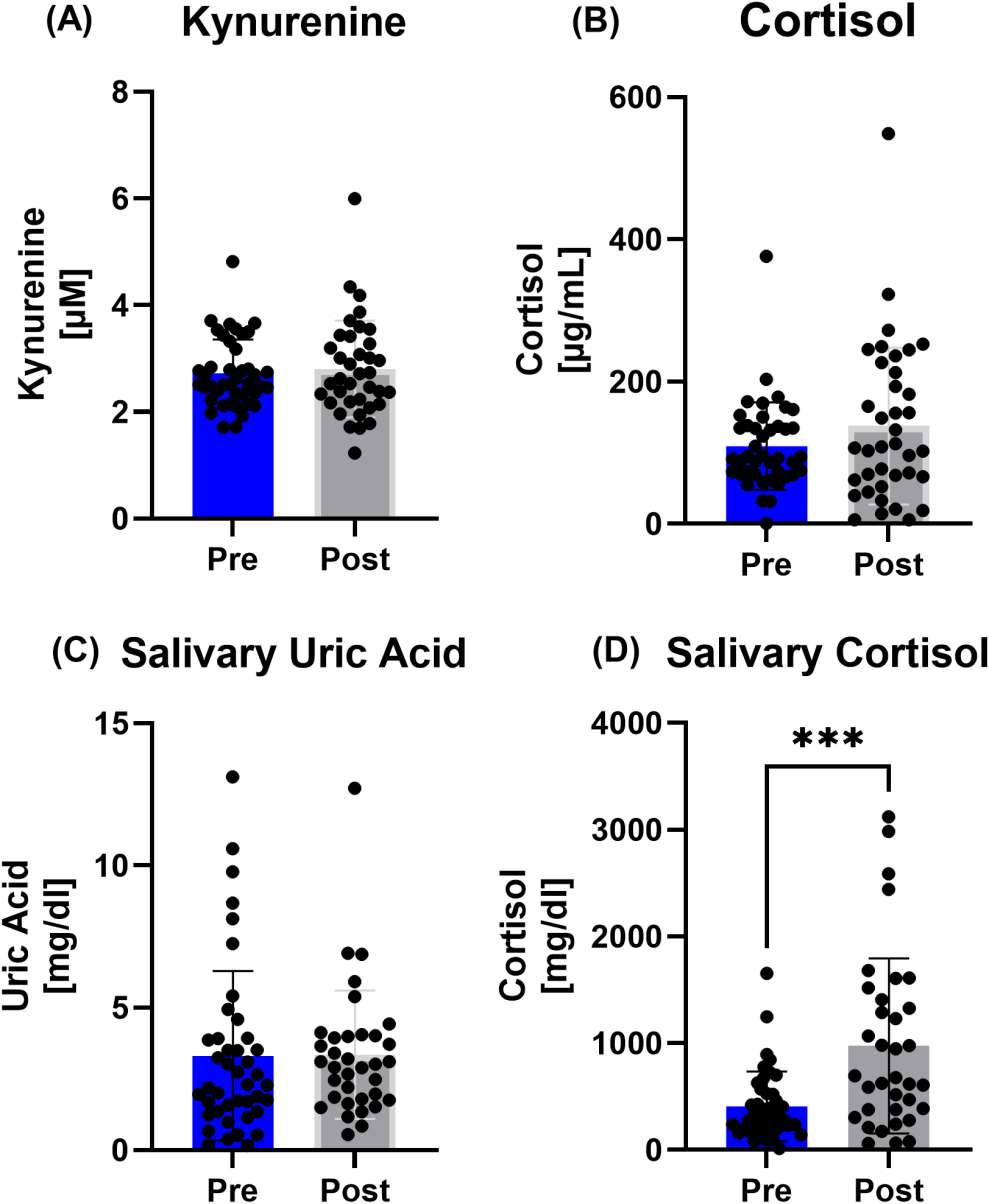
Data on **(A)** kynurenine, **(B)** cortisol, **(C)** salivary uric acid, and **(D)** salivary cortisol concentrations for all participants. While there was no difference observable in kynurenine, cortisol and uric acid Pre to Post race in all participants, salivary cortisol increased after the race. ***p < 0.001.

While there was no observable difference between Pre-Post in the kynurenine, cortisol or salivary uric acid concentrations, an increase in the stress marker salivary cortisol (p=0.0005) could be measured in the whole group (**Figure 4**).

**Figure 5:**
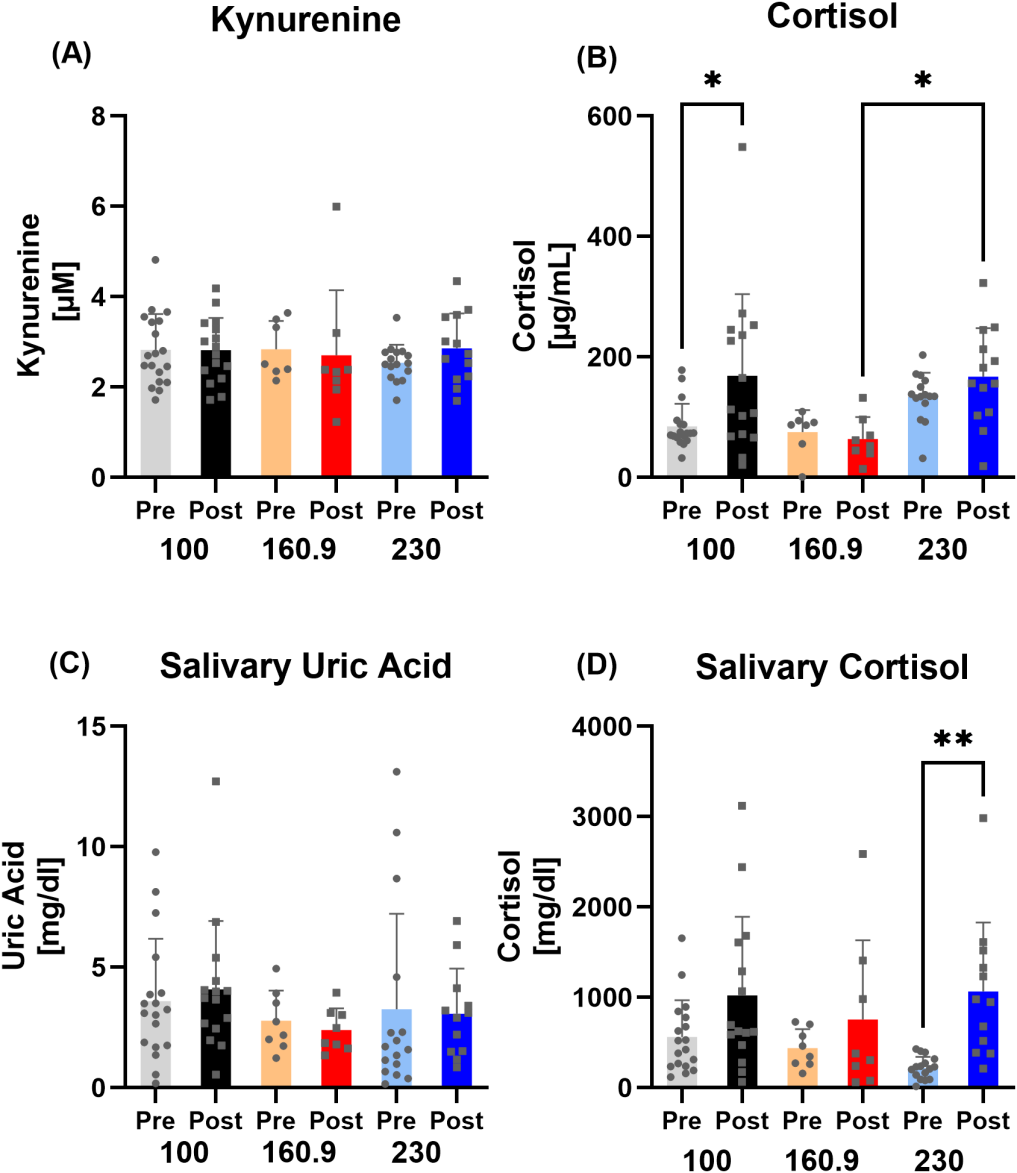
Data on **(A)** kynurenine, **(B)** cortisol, **(C)** salivary uric acid, and **(D)** salivary cortisol concentrations subdivided into 100/160.9/230 km running distance. In plasma, cortisol levels increased in 100 km runners, while there were significant higher values measurable in 230 km runners compared to 160.9. In saliva, cortisol levels increased in 230 km runners compared to the other groups Post race. *p < 0.05; **p < 0.01.

Subgroup analyses showed that plasma cortisol (p=0.0421) and saliva cortisol (p=0.0058) of the 230 km group exhibited different values Post race, while only plasma cortisol was elevated in the 100 km runners (0.0325) (**Figure 5**).

### 3.5 GASE

**Figure 6:**
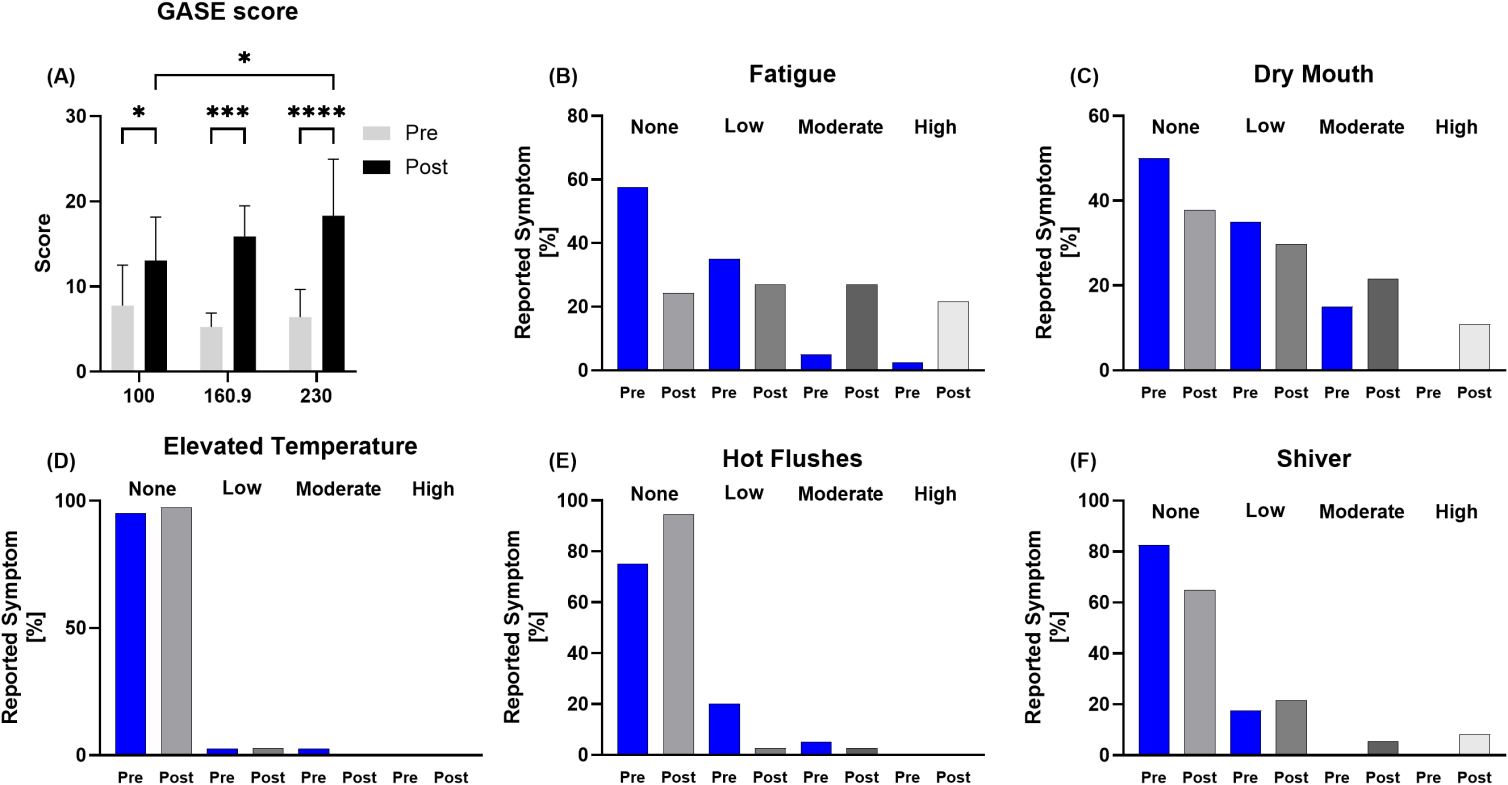
Data of the General Assessment of Side Effects (GASE, German version) questionnaire. **(A)** is showing the total score Pre-Post of the different distances, with increasing values from 100 km to 230 km runners. Regarding the immune related sub-scores **(B)** fatigue, **(C)** dry mouth, **(D)** elevated temperature, **(E)** hot flushes, and **(F)** shiver for all participants, only the relative difference Pre-Post for the rating none to high is displayed. Here, especially in the categories fatigue, dry mouth and shiver newly occurred symptoms ranging from low to high were reported. *p < 0.05; ***p < 0.001; ****p < 0.0001.

GASE determined the subjective individual side effects recognized by the runners Post race. As shown in figure 6 A, side effects increased with increasing running distances. Analysis of potential side effects related to immune function revealed an increase in symptoms, particularly in the subcategories of Fatigue and Dry Mouth. Only 2.7-5.4% of the participants experienced Elevated Temperature or Hot Flushes Post race, without reaching any high severity, whereas one third of the runners reported a type of low-high Shiver feeling Post race (**Figure 6**).

### 3.6 Exploratory analysis of possible sex differences

Sex-related differences after biomarker analysis could not be detected. Respective increases or decreases reflected the immune responses of the whole group analysis (**Supplementary Figures 1–S3**).

## 4 Discussion

Although the beneficial long-term effects of continuous endurance training on the immune system is well known (25), strenuous running competitions can lead to acute attenuation of the immunological response and an increased risk for subsequent infections (7). Running in competitions like ultramarathon events where the participation numbers are perpetually increasing (+1676% from 1996–2018) (26, 27), and which do not have any upper limit, resulting in races from 50 km up to transcontinental competition, may induce immunological alterations currently unknown. Here, the immediate or long-term effects of this strenuous exercises on the immunological response are still largely unexplored and need further investigation.

### 4.1 Blood changes: leukocytes and thrombocytes

Alongside the leukocyte increase in all groups (5, 6), an elevation in thrombocyte count was detected. These changes reflect the body’s acute adaptive response to prolonged physical stress. Hemoconcentration and redistribution are common physiological responses during long-distance running, resulting in plasma volume shifts (e.g. by fluid loss through sweating and redistribution) and/or by dehydration leading to relative increases in circulating blood cells (28). The platelet increase is driven by sympathetic activation, fluid shifts, and inflammatory mediators—all part of maintaining hemostatic balance and preparing for potential initiated tissue repair Post race (28). In another part of this study focusing on hemorheological changes during the ultramarathon, our study group showed that no changes from pre- to post-run in total blood volume, plasma volume, or red blood cell volume occurred. We therefore concluded that hemoconcentration was unlikely (24), and that the increases in leukocyte and thrombocyte concentrations that we observed were due to acute physiological responses rather than to mere changes caused by fluid shifts.

Shin and Kim observed significantly increased white blood cell count and also hs-CRP levels and following a 100 km, a 308 km and a 622-km race (29). As one of only few studies that determined the distance-related effects of ultramarathon running, they could show that the increase in the respective immune responses was more pronounced the longer the running distance lasted, confirming our observations regarding CRP and leukocytes. Leukocyte counts typically increase acutely during and immediately after exercise, driven by mobilization from the spleen, bone marrow, and marginal pools under the influence of catecholamines and stress hormones such as cortisol. Although the values also increased after 100 and 160.9 km, the increases were not significant, so that the increase in leukocytes could also be due to changes in blood composition or the induced inflammatory response (30).

In addition, cytokines actively regulate leukocyte behavior: IL-6 promotes neutrophil mobilization into the circulation, while IL-1 and TNF-α modulate leukocyte adhesion and recruitment to tissues. Conversely, activated leukocytes themselves release cytokines, creating a transient systemic inflammatory state. The timing of these responses is critical: immediately post-race, both leukocyte numbers and proinflammatory cytokines peak, whereas in the following hours leukocyte counts may decline below baseline while anti-inflammatory cytokines remain elevated.

Other studies showed that longer distances, such as ultramarathons of 100 km or more, intensify these responses, partly due to muscle damage and fatigue, and may temporarily impair leukocyte function despite high circulating numbers, reflecting a period of transient immunosuppression (31–35). Unfortunately, it was not possible to take follow-up measurements to observe possible transient immunosuppression in our study population. However, these measurements would provide valuable insights into the immune system’s response in the hours or days following running.

### 4.2 Inflammation response

The observed IL-6 increase Post race is in line with current evidence (16, 36). A study by Steensberg et al. showed that physiological IL-6 levels can trigger an anti-inflammatory response in humans by increasing both IL-1ra and IL-10, independently of TNF-α (37). IL-6 also raises cortisol, leading to neutrocytosis and late lymphopenia with similar timing and effects as seen during exercise, highlighting muscle-derived IL-6’s key role in exercise-related leukocyte movement (37). We also could observe concomitant IL-6 and IL-10/IL-1ra changes, as all were elevated after the race. Here, the effect was observed in all sub-groups, assuming a distance-independent inflammatory response to an ultramarathon. As we did not take muscle biopsies, the IL-6 contribution as a myokine remains speculative. IL-6 is stored in vesicles in skeletal muscle fibers and released in response to lactate and protease activity (38). Additionally, IL-6 is now considered an energy sensor that responds to low glycogen levels and stimulates hepatic glucose production and promotes lipolysis (39, 40). Nevertheless, assuming severe muscular damage during and after the race showed by John et al. (23) and based on data of previous studies from other (8, 36) and our group (2), the leakage of muscle derived cytokines into the circulation due to active and passive transport cannot be ruled out.

Skinner et al. also examined the immune response comparing different trail running distances. Their study compared a sub-marathon 40 km trail and a 171 km ultra-trail race regarding leukocyte counts and cytokine levels. Both races increased leukocyte concentrations and showed higher post-race IL-6, IL-1β, MCP-1, and IFN-γ. While both races raised IL-6 and IL-8, only the 171 km event significantly elevated MIP-1β, IL-7, IL-17a, and IL-4, highlighting that the longer race caused more pronounced changes in cytokine profiles (41). Although we also only assessed cytokines pre and post-race, our design may assume that cytokine increases happen fast after start and remain elevated at a similar level. Baseline as well as immediate post-race levels were comparable between all groups, suggesting that anti-inflammation initiated during the race remains activated at a high level and during the whole running time of either around 10 hours (100km), 20 hours (160.9 km) or 30 hours (230 km): a steady-state between inflammatory and anti-inflammatory orchestration seems sensible. In contrast, pre-to post differences in TNF-α^®^ and salivary IL-17a could not be measured, reflecting apparently unchanged or delayed responses to the strenuous ultramarathons. Here, further post-race follow-up measurements (e.g. 30 to 180 minutes) might give valuable insights in future studies.

A common challenge in interpreting interleukin increases in non-pathological contexts—such as exercise-induced muscle damage—is that inflammation cannot be classified per se as either “beneficial or detrimental” (42). Inflammation is increasingly recognized as central to muscle repair and regeneration. Following exercise-induced damage, intramuscular inflammation is a controlled process that supports adaptive change and restoration of normal function (42). Thus, the observed increases in the study of Skinner et al. and our study may indeed reflect both: running distance related muscle damage with possible increased inflammation, intertwined with an interleukin signal cascade to induce muscle repair.

Interestingly, IL1-beta concentrations in saliva decreased Post race, although a pro-inflammatory response may be expected. This may be explained by the concomitant increased cortisol and IL-10 concentrations, which both suppress IL1-beta and initiate an anti-inflammatory counter-response. Although saliva concentrations, as a caveat, may not reflect systemic concentrations for some biomarkers, accumulating evidence shows that saliva is capable of identifying various stressors and may be regarded as the future in field studies with athletes (43). Significant increases in salivary IgA and antimicrobial proteins concentration have been observed with acute exercise whereas conversely, decreases have been reported during a longer over a training season leaving the athlete susceptible for URTI (44), underlining the potential use of immunological stress during different exercise intensities and durations (43–45). Unfortunately, we could not measure IgA concentrations in our cohort, limiting our conclusions of possible long-lasting detrimental effects.

After sub-group analysis, IL-6, IL-10 (except for 100 km) and IL-1ra remained significantly elevated in all groups Post race. This initiation of the exercise induced inflammatory cascade thus seems independent of the running distance, with marked pro- and anti-inflammatory changes. As there were no differences between the groups, the magnitude of the respective running duration on the inflammatory response can only be speculated as no follow-up measurements for a kinetic analysis were possible. Alves et al. examined the effects of ultramarathon, marathon, and half-marathon races on cytokine levels (14) and concluded that there is a clear dose-dependent relationship with running volume: the longer the exhaustive exercise lasted, the longer the inflammatory system needs to return to normal. Underlining this, we observed increased CRP levels post-race, with the 230 km subgroup exhibiting the most pronounced elevations, which furthermore indicates increased immune system activation with increasing distance. In contrast to our results with the strongest CRP response in the 230 km subgroup, a study by Tauler et al. who compared pre- to post-race CRP in athletes competing over 25 km or 104 km (46), no association between CRP levels and distance could be found. This difference can be attributed to the different competition distances (25/104 km vs. 100/160.9/230 km) and environmental factors (separate competition days vs. one track with different distances), thus limiting a general conclusion of the CRP response in relation to race distance.

### 4.3 Stress response changes

Post-race analysis of the stress response revealed higher salivary cortisol, increasing with race distance, reflecting the highest physiological stress of the 230 km group relative to the shorter-distance subgroups, likely attributable to the cardiovascular and metabolic challenges of the race. Since Pre values were comparable between the sub-groups, circadian variation does not seem to influence the observed initial values. This might be explained by additional factors influencing cortisol levels including food intake, stress or physical activity (47, 48). This finding is in line with the observations of Hohl et al., who examined cortisol levels after a 24-h ultramarathon in experienced and inexperienced runners (49), and with those of the above mentioned study by Tauler et al., which also assessed salivary cortisol before and after a 25 km and 104 km run. In both studies, a higher concentration of cortisol was determined with increased running distance as well as speed, suggesting cortisol as a possible marker for performance level and finish time. The aforementioned studies underline the similar and possible accumulative response of cortisol with increasing running distance, irrespective of biomatrices or training adaptation. Conversely, we observed differences in the subgroups, with the strongest plasma cortisol increase in the 100 km runners, while salivary cortisol increased most in the 230 km runners. These may be due to the faster running pace with higher cortisol release for regulating energy metabolism (100 km) or the accumulated running stress and sleep deprivation (230 km). Nevertheless, as cortisol release is sensitive to night-day-fluctuations and as we only assessed pre to post-race values at different starting times in the day without controlling for salivary flow or quality, cortisol changes especially in saliva have to be interpreted cautiously in this study.

Salivary uric acid, a biomarker for oxidative stress, neither changed in the whole group nor in the subgroups pre to post race. Research on serum uric acid concentrations with regard to marathon and ultramarathon running revealed different results. While the serum uric acid levels rose slightly but significantly both after a marathon and a 100 km ultramarathon (50), long term ultra endurance exercise showed unchanged or even decreased values: during a 1600 km running event, uric acid concentrations remained stable after 4 days, slightly decreased at day 11, and changed back to baseline values immediately after the race (51). These results suggest that uric acid levels depend on the acute situation and the running distance, and may be highly interindividual even in serum. As we assessed uric acid in saliva, and although a linear relationship between serum and salivary uric acid is assumed, data on salivary uric acid in connection with longer endurance exercise is limited. Gonzalez et al. examined female and male runners before and after a 10 km race and found increased salivary uric acid concentrations after the run (52), whereas there are no data in ultrarunning research. Nevertheless, as mentioned before, our observed values pre and post-race also showed rather individual responses which did not seem to depend on running distance. Thus, more research is needed in combination with additional oxidative stress markers to establish uric acid as potential exercise stress marker in saliva.

Then again kynurenine, known as marker for a disturbed immune system and mental health (53), was unchanged compared to Pre values. As a key player in the tryptophan metabolism, kynurenine is also proposed as potential marker for overtraining and thus long-term chronic stress and inflammation (18). Chronic exposure to stressors, e.g. fast increases in training volume and competition rate, may lead to a manifestation of elevated kynurenine levels and immunological stress (54). The unchanged concentrations in our study led to the assumption that the single long-term high volume, but low intensity running (in relation to heart rate) did not manifest itself in a disturbed tryptophan metabolism, especially in well-trained athletes. The well-trained performance status of our participants had to be confirmed beforehand by a physical check of general health and year-long experience in ultramarathon training and competition, ensuring the absence of negative effects during the race (entry eligibility).

The general good training status required for completion of all distances between 100–230 km, but without competing on (inter-)national level, in combination with unchanged kynurenine and uric acid levels in all groups led to the assumption that our participants – despite acute inflammation and a strong immune response – did not exhibit any signs of chronic stress or overtraining. This is reflected by the mean completed finishes of 38 ultramarathons per runner, and the relatively low training volume of 71 km per week (comparable to marathon runners). As there was also no difference between the subgroups observable regarding kynurenine and uric acid, an intensity as well duration dependent change may be ruled out.

In contrast, the 230 km runners also showed more adverse subjective symptoms compared to the 100 km and 160.9 km runners, determined by the GASE questionnaire. The total symptom score increased with running distance and was highest for 230 km Post race. This subjective assessment of the runners themselves in conjunction with the objective measurements of saliva cortisol/CRP, the elevated cytokine response and the blood values (leucocytes, thrombocytes), underline the additional strain in comparison to the other ultramarathon durations between 15 (100 km) to 23 hours (160.9. km). Regarding the sub-scores possibly related to immune function, most differences Pre to Post were found in the categories Fatigue and Dry Mouth. Ultramarathon elicits both physiological central fatigue (greater in single stage races) and peripheral fatigue (greater in multistage races) (55), but also increases subjective fatigue while decreasing vigor and tension after a race (56). Notably, these mood disturbances can last between days up to one month post-race (56). As fatigue may be an expected and normal side effect of up to 32 hours of non-stop running like in our study, the imbalance of sufficient regeneration and the next bout of exercise exacerbates the risk for infections - especially in conjunction with a dry mouth and shivering, indicating an improper hydration status as well as insufficient water intake for normal thermoregulation. A dry mouth can contribute to less resilience against microbial infection and consequently enhance the risk for URTI. A study by Gill et al. found decreased saliva flow rate, antimicrobial salivary IgA and lysozyme secretion rates after a 24-h continuous overnight ultramarathon, but fortunately no incidences of upper respiratory symptoms were reported by their participants, albeit in-race symptoms like fever, cold-flu and headaches (57).

Our exploratory analysis of possible sex differences did not reveal any difference between female and male participants regarding our analysed biomarkers. Although a different immune response to pathogen infection and systemic autoimmunity as well as immune cell composition and function between women and men is known (22), at least in our study the respective immune and stress responses to the ultrarunning appeared similar.

Taken together, regarding the immunological, inflammatory and subjective stress response, distance and duration indeed seem to matter in ultramarathon competition, and physiological adaptations occur even after 24 hours of non-stop running. Thus, special emphasis should be on recommendations for these runners for a well-planned post-race regeneration strategy, involving evidence-based methods like reduced physical activity/training, sufficient sleep, and the fast replenishment of energy (balanced macro- and micronutrient intake) in the weeks after the race. Although an acute immune response after ultramarathon cannot be totally prevented, and probably should not be due to molecular, immunological and muscular adaptations, the runners seem at greater infection risk and follow-up immune system disturbances with increasing running duration and distance.

## 5 Strength & limitations

The presented study includes data of 43 experienced ultramarathon runners of a relatively homogeneous running performance level, which is a comparably high number in this population with a sound statistical power. Nevertheless, especially with regard to the examined biomarkers, only Pre and Post sampling, and, due to organizational constraints, no follow-up examinations could be performed. Thus, possible peaking or temporary decline of interleukins or other markers could not be determined and may have been present during the race. Sleep deprivation/running during the night may also alter the biochemical response, although all runners had the same conditions here.

Although the statistical power declines with sub-group analyses (multiple testing and smaller sample size), but to the best of our knowledge there is no other study that compared the examined ultramarathon distances during one race, on the same track and under comparable conditions. In addition, the combination of a wide range of biochemical analyses in blood as well as saliva and the subjective assessment of the validated GASE questionnaire adds further data to mechanistic immunological insights induced by these ultra-endurance competitions.

## 6 Conclusion

Distance and duration determine the impact of the immunological response regarding the examined biomarkers and subjective measurements. Although ultramarathon training per se seems safe and does not lead to chronic stress or inflammation, non-stop ultra-distance running competitions induce acute changes in the pro-inflammatory cytokine response with concomitant counter-reactions of anti-inflammatory biomolecules. The long-term effects or the duration of these change remain speculative without follow-up measurements in the following hours/days, but should be regarded when considering duration and intensity of ultramarathon events.

## Data Availability

The raw data supporting the conclusions of this article will be made available by the authors, without undue reservation.
