# Supplementary material for: (Ultra-)inflammation or adaptation? Comparison of different ultramarathon distances and their effect on the immune system
Source: Front Immunol. 2026 Apr 13;17:1799887. doi: 10.3389/fimmu.2026.1799887 (PMC13112676; doi:10.3389/fimmu.2026.1799887)
Supplement: Supplementary file 1 [file DataSheet1.docx]

**Supplemental**

Figure S1: Data on (A) leukocyte concentration and (B) thrombocyte concentration for female ♀ and male ♂ participants. While there was an increase in leucocytes Post race in both sexes, thrombocyte cells did only differ between Pre and Post. ****p < 0.0001.

Figure S2: Data on (A) interleukin 6 (IL-6), (B) interleukin 10 (IL-10), (C) tumor-necrose-factor-alpha (TNF-alpha), (D) interleukin 1ra (IL-1ra), (E) salivary C-reactive protein (CRP), (F) salivary interleukin 1-beta (IL1-beta), and (G) salivary interleukin 17a (IL17a) concentrations for female ♀ and male ♂ participants. While salivary IL1-beta decreased Post race in all participants, the interleukins IL-6, IL-10 and IL-1ra as well as salivary CRP increased after the race. *p < 0.05; **p < 0.01; ***p < 0.001; ****p < 0.0001.

 Figure S3: Data on (A) kynurenine, (B) cortisol, (C) salivary uric acid, and (D) salivary cortisol concentrations for female ♀ and male ♂ participants. While there was no difference observable in kynurenine, cortisol and uric acid Pre to Post race in all participants, salivary cortisol increased after the race in men. *p < 0.05.
